# Polyphenols and post-exercise muscle damage: a comprehensive review of literature

**DOI:** 10.1186/s40001-025-02506-6

**Published:** 2025-04-09

**Authors:** Xiaofeng Zhang, Yuanfu Zhong, Sogand Rajabi

**Affiliations:** 1 Yinchuan University of Energy, Yinchuan, 750105 Ningxia China; 2 School of Sports and Health Sciences, Xiangsihu College of Guangxi Minzu University, Nanning, 530000 Guangxi China; 3 Department of Cellular and Molecular Biology, Islamic Azad University, Sirjan Branch, Sirjan, Iran

**Keywords:** Exercise, Polyphenols, Injury, Inflammation, Fatigue

## Abstract

Recent research highlights the significant role of polyphenols in alleviating post-exercise muscle damage, thus positioning them as a valuable nutritional strategy for athletes and fitness enthusiasts. Polyphenols, naturally occurring bioactive compounds abundant in fruits, vegetables, tea, wine, and other plant-based foods, are recognized for their potent antioxidant and anti-inflammatory properties. This dual mechanism is critical for combating oxidative stress and inflammation—two factors that intensify during vigorous physical activity and contribute to muscle soreness and damage. Among various polyphenols, compounds like quercetin have particularly emerged as effective agents for promoting muscle recovery and enhancing exercise performance. These protective effects are facilitated through several mechanisms, including the modulation of inflammatory pathways, acceleration of muscle repair processes, and enhancement of mitochondrial function, all of which bolster overall muscle health. As ongoing studies yield deeper insights, the potential of polyphenols to enhance athletic performance and overall health will become increasingly substantiated, leading towards their strategic incorporation into exercise nutrition protocols. Therefore, we reviewed relevant studies in order to show how efficient polyphenols can be in reducing muscle fatigue and damage and what are the exact mechanisms.

## Introduction

Polyphenols are a class of phytochemicals which can be extracted from a variety of plants, fruits, and vegetables. Red wine, dark chocolate, tea, and berries are some of the best-known sources. Yet, many other foods also offer significant amounts of these compounds. Polyphenols are believed to serve a variety of roles in plant ecology, including the release and inhibition of growth hormones like auxin, acting as UV filters to shield against ionizing radiation and contributing to coloration, serving as deterrents to herbivores through their sensory properties, and protecting against microbial infections as phytoalexins. Recently, these natural compounds have been of an interest among the scientific community due to their many beneficial characteristics including anti-inflammation and anti-oxidant effects [1–3].

Skeletal muscle is the most prevalent type of tissue in the human body. A key feature of skeletal muscle is its ability to recover after an injury, regardless of the injury's cause, through a process known as the inflammatory response. These injuries are very prevalent among athletes. The types of common injuries include contusions, hematomas, myositis ossificans, delayed-onset soreness, strains, rhabdomyolysis, and lacerations [4]. The most common mechanisms which cause muscle injury are trauma and contraction, especially eccentric contractions. These injuries cause a disruption in muscle function leading to a reduced range of motion in the joint, changed levels of fatigue, and a slower muscle shortening speed; however, the most commonly acknowledged consequence is the ongoing loss of strength [5]. Since strength loss caused by injuries can have a prolonged effect on performance at work, in sports, and at home, it is essential to provide a safe and cost-effective method for either preventing these injuries from happening or decreasing the levels of strength loss after an injury happened.

In this review, we have tried to gather as much as possible information in order to answer the questions about the efficacy of polyphenols in either preventing muscle injury or attenuating muscle damage and also, the mechanisms by which these natural products exert their effects. We would also take a look into the efficacy of polyphenols in enhancing muscle performance, and recovery after exercise.

## Polyphenols

**Fig. 1:**
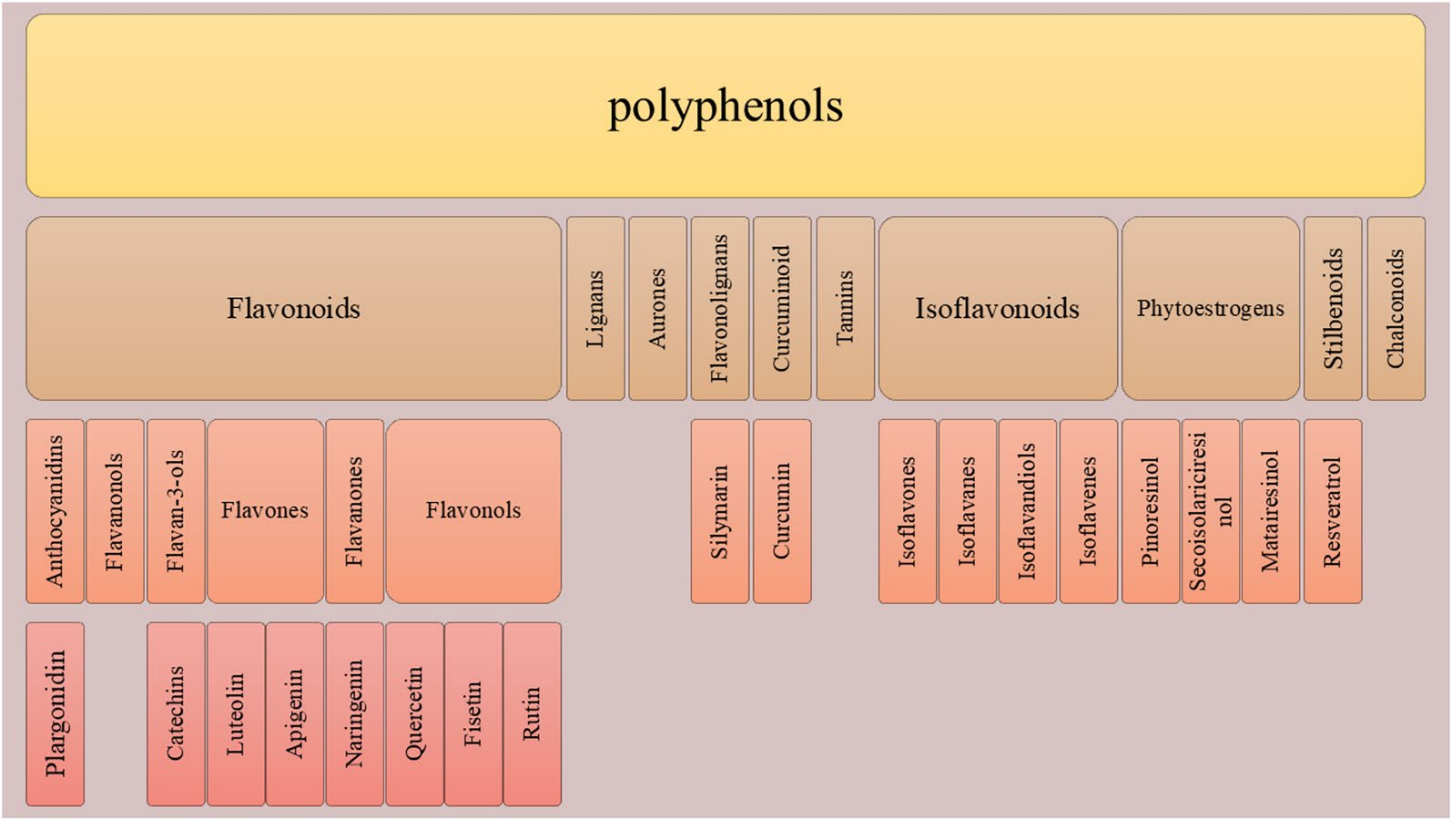
Classification of polyphenols and some examples of each class

The term ‘phenol’ describes a chemical structure that consists of an aromatic benzene ring attached to a hydroxyl (− OH) group, which is why it has the -ol suffix. Polyphenols are named after their structure which contains several phenol groups. phenolic acids, flavonoids, stilbenes, and lignans are the main subgroups of this superfamily however, there are other members in this family such as tannins, aurones, chalconoids, phytoestrogens, etc. (all shown in Fig. 1) [6, 7].

### Flavonoids

Flavonoids or bioflavonoids, derived from the Latin term ‘‘flavus’’ meaning yellow—reflecting their natural coloration—belong to a group of polyphenolic secondary metabolites present in plants and are regularly included in human diets. In terms of chemistry, flavonoids feature a basic structure consisting of a 15-carbon backbone made up of two phenyl rings (referred to as A and B) and a heterocyclic ring (C), which contains an embedded oxygen atom. This carbon arrangement is commonly represented as C6-C3-C6. Flavonoids are a great subclass of polyphenols which contain many variations given their structures [6, 7]. According to International Union of Pure and Applied Chemistry (IUPAC) naming conventions. Flavonols are a type of flavonoids characterized by the 3-hydroxyflavone structure (IUPAC name: 3-hydroxy-2-phenylchromen-4-one). Their variation arises from the different positions of the phenolic –OH groups. Flavonols are different from flavanols, like catechin, which belongs to another flavonoid category, as well as from flavins which are unrelated metabolically significant compounds derived from the yellow B vitamin riboflavin. Quercetin, rutin, fisetin, and kaempferol are some examples of flavonols and are commonly found in apples, berries, and wine [7].

Flavones are another line of flavonoids which are either colorless or yellow compounds that are derived from the naringenin flavanone by the introduction of a double bond to the C-ring between carbon atoms 2 and 3. They have a structure that is quite similar to that of flavonols, with the main difference being the lack of hydroxyl groups at the 3-position on the C-ring. In the majority of higher plants, flavones are produced from unconjugated flavanone intermediates through the activity of flavone synthases (FNSs) [8]. Flavones are not as prevalent as flavonols in fruits and vegetables. They are primarily made up of glycosides of luteolin and apigenin. In contrast, polymethoxylated flavones like tangeretin, nobiletin, and sinensetin are found in significant quantities in citrus peels. There have been only a limited number of flavone sources identified [8]. acacetin, chrysin, tangeritin, and diosmetin are some other examples of flavones. Flavanones have the structure of a benzopyranone core that is modified at the C2 position, with potential alterations to the aryl backbone of this core [9]. In nature, chalcone isomerase (CHI) facilitates the transformation of 2′-hydroxychalcones into (2S)-flavanones, while various enzymes carry out the conversion of flavanones into other metabolites found in plants [10]. Hesperidin, naringenin, silybin, and eriodictyol are some examples of this subclass [10]. Flavanols which contribute to plant defense are found in most plants. These are derivatives of flavans characterized by a 2-phenyl-3,4-dihydro-2H-chromen-3-ol framework. Flavan-3-ols exhibit structural diversity and encompass various compounds. catechin, epicatechin gallate, epigallocatechin, epigallocatechin gallate (EGCG) are some of the most known types of this class [11]. Besides flavonoids which have attracted great attention, other subgroups of polyphenols are also known to have antioxidant effects and other advantageous characteristic.

### Curcuminoids

**Fig. 2:**
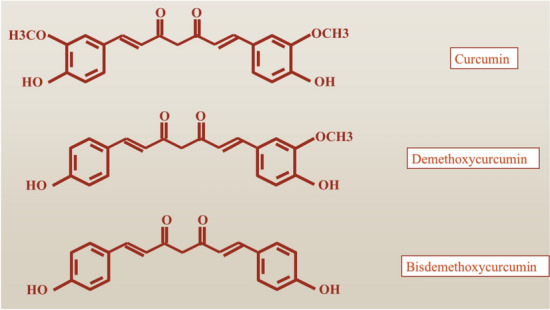
Three main forms of curcumin include: demethoxycurcumin (DMC) and bisdemethoxycurcumin (BDMC) and curcumin (CUR)

Curcuminoids, particularly curcumin, are a group of polyphenols which are the main components found in the Indian spice turmeric, which is derived from the rhizomes of the *Curcuma longa* plant, a member of the ginger family [12]. This plant has been utilized for medicinal purposes for many years, predominantly in Asia. These agents are linear, diarylheptanoid molecules that include curcumin and related compounds. The turmeric plant *C. longa* naturally contains two primary forms of curcumin: demethoxycurcumin (DMC) and bisdemethoxycurcumin (BDMC) (the structure of these forms is shown in Fig. 2) [12, 13]. Additionally, a rarer variant known as cyclocurcumin is also naturally present in *C. longa*, although in smaller amounts. Furthermore, within the human body, curcumin is quickly metabolized into various byproducts, including hydrocurcumin (which can exist in saturated forms like di-, tetra-, or hexa-hydrocurcumin), glucuronides, and sulfated curcumin [12, 13].

The compound curcumin (CUR), also referred to as 1,7-bis-(4-hydroxy-3-methoxyphenyl)-hepta-1,6-diene-3,5-dione and with the chemical formula C21H20O6, is the most extensively researched and is the predominant polyphenol found in turmeric [14]. Curcumin’s use is mainly restricted due to its unfavorable pharmacokinetic and pharmacodynamic characteristics, which include limited absorption, a short half-life, and quick metabolism in the gastrointestinal system. The Joint FAO/WHO Expert Committee on Food Additives and the European Food Safety Authority recommend a daily intake of curcumin ranging from 0 to 3 mg per kilogram [14]. A great body of evidence has shown that curcumin has many molecular effects and is involved in several cellular processes such as histone modification [15], cell survival [16, 17], inflammation [16, 17], and oxidative stress [17]. Given these advantageous effects of this natural product, it is used for treating many diseases including diabetes [18], obesity [18], neurodegenerative disease [19], and cancer [20].

### Stilbenoids

**Fig. 3:**
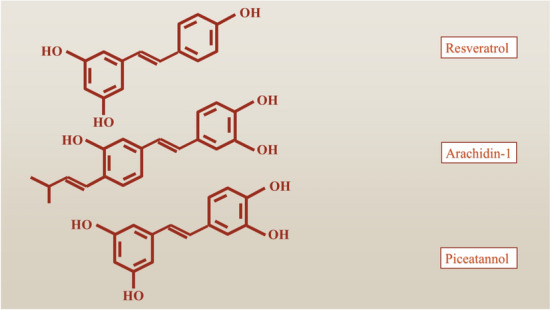
A schematic presentation of the structures of the most common stilbenoids including resveratrol, arachidin-1, and piceatannol

Stilbenoids are a fascinating class of polyphenolic compounds featuring a core stilbene structure, characterized by a biphenyl framework with a double bond connecting two aromatic rings (the structure of most known stilbenoids are shown in Fig. 3) [21]. These compounds have gained significant interest due to their wide range of biological activities and their potential health benefits. The most well-known stilbenoid is resveratrol (RSV), which is found in various plants, particularly in the skin of grapes, and has been extensively studied for its antioxidant properties and cardiovascular benefits [21].

Stilbenoids are predominantly produced by plants as a response to environmental stress, including UV radiation, pathogen attacks, and physical injuries [22]. The production of these compounds is part of a larger class of plant secondary metabolites that serve crucial roles in plant defense. Beyond resveratrol, other members of this family include pterostilbene, which has garnered attention for its potential anti-cancer and anti-inflammatory properties, and other less well-known stilbenoids like ε-viniferins, which are often found in red wine and have been linked to various health benefits [22, 23].

Research continues to unveil the myriad ways in which stilbenoids interact with biological systems [24]. For instance, resveratrol has been shown to activate certain sirtuins, a family of proteins involved in cellular regulation, which may have implications for aging and metabolic health. These compounds also possess anti-inflammatory, antiviral, and neuroprotective properties, suggesting their potential as therapeutic agents in managing various diseases, including cancer, diabetes, and neurodegenerative disorders [24].

The dietary sources of stilbenoids are diverse, with notable concentrations found in red wine, peanuts, berries, and certain herbs. This naturally raises interest among consumers and researchers alike regarding the potential health benefits of including stilbenoid-rich foods in the diet [22]. RSV is mostly observed in grapes, nuts, and peanut while Piceatannol and Pterostilbene are only found in grapes and arachidin-1 and −3 is only observed in peanuts. However, the bioavailability and metabolism of these compounds in the human body remain subjects of ongoing investigation, as they can vary significantly based on the source and the method of consumption [22].

The future of stilbenoid research promises exciting developments, particularly in the realm of dietary supplements and functional foods. Advances in biotechnology may lead to enhanced extraction and production methods, improving the availability and efficacy of these compounds. Overall, stilbenoids represent a captivating intersection of nature, nutrition, and pharmacology, highlighting the profound connection between plants and human health.

After investigating the structures, characteristics, and impacts of polyphenols, it is necessary to explain the mechanisms of muscle fatigue and damage in order to make a logical linkage between them.

## Muscle fatigue and damage

### Muscle fatigue

Muscle fatigue can be categorized into two main types: temporary and chronic muscle fatigue [25]. Temporary fatigue, which arises as a result of intense physical exertion. This condition typically occurs due to the accumulation of byproducts from energy metabolism within the muscle cells, such as lactate, or due to the depletion of energy reserves, notably glycogen stores. The duration of recovery from temporary muscle fatigue is dependent on two factors: the intensity and duration of the physical activity performed, with a general recovery period of approximately 3 to 5 days being expected. Common strategies to facilitate muscle recovery include the use of massage therapy, cold compresses, and the administration of mild analgesics. However, it is important to seek medical assistance if muscle fatigue persists for more than two weeks. Chronic muscle fatigue can be attributed to several factors: Muscle atrophy resulting from immobilization, commonly referred to as disuse atrophy, or due to chronic inflammation associated with cardiovascular and respiratory illnesses, such as heart failure and chronic obstructive pulmonary disease (COPD) in addition to trauma or medications that act as PPAR agonists. Another factor that has been associated with chronic muscle fatigue is age-related muscle atrophy which is also known as sarcopenia. Furthermore, neurogenic muscle atrophy can attribute to chronic muscle fatigue. This phenomenon occurs when there is an obstruction or disruption in the transmission of nerve signals from the central nervous system (CNS) to the motor neuron junction, often due to a disease or assaults to spinal cord. This type of fatigue can be further classified into central and peripheral fatigue [26]. Central fatigue originates in the CNS, as seen in conditions like multiple sclerosis, leading to a diminished neural drive to the muscles [27, 28]. Conversely, peripheral fatigue arises from alterations at or beyond the neuromuscular junction. This can occur in autoimmune diseases where abnormal immune responses target synaptic proteins, such as in Graves' disease, Guillain–Barré syndrome, and myasthenia gravis. Besides, it is observed in muscular dystrophies (MDs), which are hereditary conditions characterized by progressive muscle wasting and weakness. Examples of MDs include Duchenne muscular dystrophy, which is characterized by the absence of the dystrophin protein. Another example of MD is Becker muscular dystrophy, which involves a mutated dystrophin gene. Limb-girdle muscular dystrophy type IIA is also considered as an MD which is associated with a mutation in the calpain 3-P94 gene. The presence of chronic abnormal fatigue significantly impacts the functional capacity and quality of life for affected individuals, often hindering their daily activities and, in severe cases, leading to decreased survival, particularly in instances related to neurogenic muscle atrophy [25].

### Mechanisms of muscle damage

Exercise-induced muscle damage (EIMD) refers to a series of events that arises after engaging in novel or unfamiliar physical activity, especially when it involves a significant number of eccentric contractions [29]. This process of damage is typically presented by a temporary reduction in muscle functionality, which includes both the strength of the muscle and its range of motion [30, 31]. Additionally, it may present as increased swelling within the affected muscle group, heightened levels of muscle-specific proteins in circulation, and delayed-onset muscle soreness (DOMS) [30, 32, 33]. The majority of EIMD's symptoms and indicators become evident immediately following the initial exercise session and can last for as long as 14 days [34]. These factors are commonly employed to evaluate the degree of muscle damage, with DOMS being the most frequently monitored indicator. However, the precise mechanisms underlying its occurrence remain ambiguous [35, 36]. While the specific mechanisms responsible for EIMD are not fully understood, the damage can be conceptually divided into two phases: the initial or primary damage that occurs during the exercise itself, and the secondary damage phase that transpires post-exercise, which occurs due to an inflammatory response [37].

#### Primary muscle damage

Research indicates that mechanical loading during eccentric exercise is a primary cause of muscle damage, surpassing metabolic factors [38, 39]. Compared to concentric or isometric contractions, eccentric contractions activate fewer motor units at the same force level, resulting in increased stress on a limited number of muscle fibers [40, 41]. Fast-twitch fibers are particularly susceptible to damage during these contractions, as faster motor units are preferentially recruited [42, 43]. It is widely accepted that two key indicators of muscle damage appear right after a muscle undergoes several eccentric contractions. These indicators are the disruption of sarcomeres in myofibrils and harm to the excitation–contraction (E-C) coupling system. However, there is ongoing debate about which of these two is the main event [44].

The uneven lengthening of sarcomeres can lead to structural failure, notably the “popping” of some sarcomeres, which further stresses passive structures and causes deformation of non-contractile proteins [39, 44]. During an active lengthening, longer and weaker sarcomeres become stretched onto the descending portion of their length–tension curve. Here, they lengthen quickly and uncontrollably until they exceed the overlap of myofilaments, stopping further lengthening as the tension in passive structures prevents it. Continuous overextension of sarcomeres results in their damage. Muscle fibers that have damaged sarcomeres alongside intact ones exhibit a change in the ideal length for generating tension, leaning toward longer muscle lengths. This process exacerbates fiber disruption over repeated eccentric contractions [39, 44, 45]. The exact mechanisms of sarcomere disruption that occur after eccentric contractions are still not fully understood. This process might involve the elastic filament titin, which secures thick filaments to Z discs, or the structural protein desmin, which connects neighboring Z discs [46]. It is possible that minor alignment mistakes could cause the thick and thin filaments of overstretched sarcomeres to collide. Additionally, the inactivation of certain sarcomeres due to damage to t-tubules might also contribute to this issue [46].

Additionally, when the process of excitation–contraction (E-C) coupling fails, it leads to the muscle damage, evidenced by reduced force production and diminished sarcoplasmic reticulum Ca2 + release following eccentric exercise [35, 47, 48].

#### Secondary muscle damage

Following the initial phase of muscle injury, an influx of calcium ions (Ca2 +) into the cytoplasm contributes to further damage. Elevated intracellular Ca2 + results in the degradation of structural proteins by activating proteolytic and phospholipase A2 pathways [49, 50]. While mitochondria attempt to maintain calcium homeostasis through uptaking this ion, excessive Ca2 + can result in mitochondrial dysfunction, potentially triggering cell death [49]. This excess calcium may also lead to uncontrolled muscle contractions [45, 46]. The inflammatory response that follows is crucial for clearing damaged tissue and facilitating repair [51]. Various immune cells, including neutrophils and macrophages, infiltrate the affected area in a coordinated manner. Neutrophils are typically the first responders, activated by elevated Ca2 + and releasing pro-inflammatory cytokines [52]. They help clear necrotic fibers but can also release cytotoxic substances that may exacerbate damage [53]. Conversely, macrophages initially promote degradation but can shift to an anti-inflammatory role, releasing growth factors that aid recovery [54].

#### Satellite cell involvement in muscle repair

Muscle fibers have limited regenerative abilities, relying on satellite cells, which are located between the sarcolemma and its basal lamina [55]. These cells remain inactive until stimulated by various signals, including inflammatory factors, intracellular signaling, and interactions of extracellular matrix components [56]. Research shows that intense eccentric exercise significantly activates satellite cells, leading to increased muscle regeneration [35, 57]. Satellite cells play a crucial role in the remodeling of untrained skeletal muscle, likely to ensure an appropriate ration of DNA to protein. Current research predominantly involves non-trained or sub-elite athletes, leaving the applicability of these findings to elite athletes uncertain. Notably, during periods of unloading or detraining, the nuclei that myofibers accumulate due to satellite cell activation can persist for as long as 60 days [58, 59]. Therefore, it is implied that prolonged activation of satellite cells equips the muscle with an enhanced ability to respond to repeated challenges to myofiber homeostasis.

Therefore, promoting satellite cell activity in response to muscle-damaging exercises may represent a potential strategy for nutritional interventions aimed at improving recovery [34].

#### The role of inflammation and oxidative stress in muscle damage

Other mechanisms by which post-exercise damage is initiated through both inflammation and oxidative stress. Many studies have indicated that oxidative stress and inflammation work in a parallel way. The involvement of superoxide (O2• −) in the inflammatory response was suggested by the anti-inflammatory properties of the antioxidant enzyme superoxide dismutase (SOD) when given by injection [60]. The harmfulness of O2• − , or the reactive species it produces, was believed to be the reason behind the anti-inflammatory effects of SOD [16] (refer to Fig. 1). Nevertheless, it has also been proposed that O2• − itself is fairly harmless, and its involvement in inflammation may not be limited to direct cell damage, but rather linked to the creation of a chemical substance that attracts neutrophils [61].

Another mechanism supported by many studies is the activation of redox-sensitive transcription factors, like nuclear factor κB (NF-κB), which influence the gene expression of crucial factors involved in inflammation, including IL-1β, IL-6, TNF-α, cyclooxygenase-2 (COX-2), adhesion molecules, and inducible nitric oxide synthase (iNOS). NF-κB is a transcription factor made up of two parts that belong to the Rel family. Upon activation, NF-κB moves into the nucleus and attaches to particular locations within the promoter regions of various genes. It can be activated by several external factors, including reactive oxygen species (ROS) and cytokines [62].

Numerous genes necessitate the binding of NF-κB to initiate their transcription. Noteworthy among these are manganese superoxide dismutase (MnSOD), iNOS, COX-2, γ-glutamylcysteine synthetase (GCS), vascular cell adhesion molecule-1 (VCAM-1), and various cytokines. These genes play crucial roles in a myriad of biological processes, including antioxidation, inflammation, immune responses, and apoptosis [63].

The work of Hollander et al. [64] first established a link between exhaustive exercise and the activation of MnSOD gene expression in the skeletal muscle of rats, which was a result of enhanced binding of NF-κB observed in muscle nuclear extracts. Their findings indicated that NF-κB could be activated in a manner sensitive to redox changes during muscle contractions, with peak NF-κB binding levels occurring approximately two hours after exercise in the skeletal muscle of rats. This led to the conclusion that reactive oxygen species (ROS) activate a series of intracellular pathways ultimately resulting in increased MnSOD gene expression. It was posited that the oxidation of cysteine residues in the activators of NF-κB, induced by ROS, could initiate an inflammatory response [64].

Strenuous exercise, particularly activities involving eccentric muscle contractions, can lead to muscle fiber damage that stimulates the release of inflammatory cytokines from both immune cells and damaged muscle tissue. Blood-borne polymorphonuclear neutrophils (PMNs) are vital in protecting against viral and bacterial infections by activating reduced nicotinamide adenine dinucleotide phosphate (NAD(P)H) oxidase, which generates ROS through a rapid metabolic response [65, 66]. During the acute phase of muscle injury, these inflammatory cytokines encourage the expression of adhesion molecules, including VCAM-1, cytokine-induced neutrophil chemoattractant-1 (CINC-1), monocyte chemoattractant protein-1 (MCP-1), and nitric oxide (NO). Furthermore, certain cytokines interact with their membrane-bound receptors, triggering specific ROS-producing enzymes, such as NAD(P)H oxidase and xanthine oxidase (XO). Damaged muscle endothelial cells are known to release cytokines like TNF-α, IL-1, IL-6, and IL-8, which promote a self-perpetuating cycle of inflammation. As such, oxidative stress and inflammation are inextricably linked [65, 66].

Maintaining glutathione homeostasis is also critical in managing muscle inflammation, with GCS activity being the primary regulator of intracellular levels of reduced glutathione (GSH). Elevated GSH concentrations can offer partial protection against inflammatory processes by suppressing the expression of intercellular adhesion molecule-1 (ICAM-1) [67]. Interestingly, it has been documented that the GSH content is higher in the tibialis muscle of rabbits 24 h following an isokinetic stretch injury, along with increased activities of glutathione peroxidase (GPX) and glutathione reductase (GR). Given that an optimal ratio of reduced to oxidized glutathione (GSH:GSSG) is vital for redox signaling, these findings substantiate a clear correlation between oxidative stress and inflammation within skeletal muscle cells [67, 68].

To further explore the interplay between oxidative stress and inflammation in muscle tissue, it is essential to understand the underlying mechanisms. Reactive oxygen species are not merely by products of cellular metabolism; they serve as signaling molecules that can modify cellular functions. The production of ROS in response to exercise can activate several pathways, including those involving NF-κB [69]. This transcription factor is instrumental in the expression of genes that mediate various physiological responses, including those linked to oxidative stress and inflammatory pathways. Moreover, the persistence of oxidative stress after heavy exercise can compromise muscle recovery and lead to long-term damage. Inflammatory responses following muscle damage are necessary for tissue repair, but if left unchecked, they can contribute to chronic inflammation, which is detrimental to muscle health. Therefore, a balance must be maintained between the beneficial and harmful effects of ROS during the recovery process [69].

The involvement of antioxidants in this balance is particularly noteworthy. Some antioxidant compounds such as vitamin C and flavonoids have been recognized for their ability to scavenge free radicals, thereby mitigating oxidative damage. Their role extends beyond mere antioxidant capacity; they can also influence signaling pathways that govern inflammation, promoting a quicker recovery post-exercise [70, 71].

In summary, the activation of various genes through NF-κB binding during and after exercise highlights a crucial aspect of cellular adaptation to physical stress. The intricate relationship between oxidative stress, inflammation, and recovery processes in skeletal muscle emphasizes the importance of maintaining redox balance. Antioxidants play a pivotal role in mediating this balance, providing a potential avenue for enhancing performance and recovery in physically active individuals. Investigating these relationships more deeply will aid in the development of targeted strategies to support muscle health and overall athletic performance.

The interplay between antioxidant supplementation, inflammation, and recovery in skeletal muscle underscores the importance of a multifaceted approach to nutrition and exercise. Understanding which antioxidants can effectively reduce inflammation and enhance recovery opens avenues for nutritional interventions to support athletes and individuals engaged in intensive exercise. Consequently, further research is essential to delineate the specific mechanisms through which these antioxidants exert their effects and to identify optimal dosages and timing in relation to exercise.

## Dietary interventions to reduce muscle fatigue and damage

Dietary patterns have been associated with multiple chronic diseases and are considered significant contributors to global mortality and morbidity [72]. A balanced diet has been shown to exert considerable positive effects on individual health, body weight, and cardiovascular well-being [73, 74]. Investigations into nutritional interventions reveal that certain foods may exhibit anti-inflammatory properties, counteracting chronic inflammation and oxidative stress, both of which are pivotal contributors to chronic pain [73, 75]. On the other hand, some dietary components may modulate immune function and pain perception, thereby improving functional limitations related to musculoskeletal disorders and enhancing overall quality of life [76, 77]. However, it is important to recognize that different foods possess unique properties and mechanisms that may alleviate pain and other musculoskeletal issues, and the underlying mechanisms of these interactions require further investigation [78]. Certain food items are regarded as potentially advantageous in alleviating musculoskeletal pain, particularly fruits, vegetables, and whole grains [75]. A number of foods and substances with functional attributes have been investigated for their anti-inflammatory properties and potential analgesic effects. Notable examples include fish oil, olive oil, turmeric, green tea, grapes, wine, peppers, cabbage, cocoa, and apples which contain omega-3 fatty acids, resveratrol, capsaicin, and flavonoids [79–90].

These investigations show that nutrition can have a pivotal role in reducing muscle fatigue and damage after exercise and therefore, a more precise look into some dietary components such as polyphenols might be helpful.

## Polyphenols and muscle damage

As mentioned, a number of antioxidants are examined on muscle damage and fatigue and polyphenols are one of them; however, there is not enough evidence for the impacts of all polyphenols and some of them including quercetin, resveratrol, and curcumin are the most studied types which will be discussed. The effects of different polyphenols in both cellular and molecular levels are discussed in this section and a summary of these effects is presented in Tables 1, 2, and 3.

**Table 1:** A summary of studies investigating curcumin’s effects on muscle injury, fatigue, and damage

Year of publication	Administration	Model of study	Result(s)	References
2023	A single daily dose for 12 weeks	Human study	Decreasing muscle fatigue score and the muscle soreness score only in men	[92]
2023	28 days intragastric administration of either curcumin (58 mg/kg) or caffeine (6 mg/kg)	Animal study	Increasing quadriceps coefficient, muscle glycogen (MG) content, and increase in the expression of Akt, AMPK, PI3K, and mTOR proteins and curcumin decreased AMP/ATP ratio and lactic acid and increased glycogen synthase and myonectin	[93]
2022	50, 100, and 200 mg of curcumin for 4 weeks	Animal study	Increasing exercise capacity and decreasing blood urea nitrogen, blood ammonia, lactic acid, creatine kinase, and lactate dehydrogenase especially in C100	[94]
2015	180 μg/mL by oral gavage at 0, 12.3, 24.6, or 61.5 mL/kg/day for 4 weeks	Animal study	Increasing exercise duration, decreasing serum lactate and ammonia and CK and AST and ALT	[95]
2007	10 mg, oral, for 3 days prior to run	Animal study	Increasing running performance through decreasing IL-6, IL-1β and TNF-α	[96]

**Table 2:** A summary of studies investigating quercetin’s effects on muscle injury, fatigue, and damage

References	Exercise modality	Treatment use	No. of subjects	Population of interest	Treatment duration	Outcomes
[97]	Western States Endurance Run	1000 mg/day	63	Humans	3 weeks	No significant changes in CRP, Ck, and interleukins
[98]	Resistance training session	1000 mg/day	10	Humans	Single dose	Enhancing the neuromuscular performance
[100]				Humans	8 weeks	
[99]	70% V̇O2max cycling for 60 min, followed by 3 h of recovery, then a subsequent single bout of cycling exercise with 75% V̇O2 max to exhaustion	1000 mg per day	12	Humans	7 days	Increasing high-intensity cycling time to exhaustion, whole-body insulin-stimulated glucose uptake and decreasing exercise-induced oxygen stress and pro-inflammation
[101]	eccentric exercise	1000 mg/day	16	Humans	14 days	Reducing strength loss and decreasing CK and LDH
[106]	Treadmill 5 days a week for 6 weeks	1000 mg	26	Humans	8 weeks	No significant change in lactate concentration, body fat percentage, or VO2 max while increasing time to exhaustion
[102]	Intensive endurance exercising	500 mg/day	60	Humans	8 weeks	Not improve exercise performance but reducing muscle damage and body fat percent
[103]	5 km running performance, and ran a 10 km race followed by 100 drop jumps	140 mg	24 women and 33 men	Humans	one hour before competition, followed by three additional doses every eight hours	Decreasing the muscle pain and loss of performance and mechanical impulse
[104]	An eccentric-induced muscle-damaging protocol	1g/day	12	Humans	14 days	Reducing myoglobin, Ck, LDH, and IL-6 and increasing IGF-I and -II
[122]	2 separate sessions of 24 eccentric contractions of the elbow flexors	1000 mg/d	30	Humans	7 days before and 5 days after the second exercise session	Plasma QUE obtained levels of 202 ± 52 ng/ml after seven days and stayed elevated throughout the 5 day recovery
[105]	Treadmill (28 m/min at 5° slope for 90 min) for seven consecutive days	100 mg/kg		Adult male BALB/C mice	4 weeks	Decreasing mitochondrial oxidative stress by inhibiting glutathione depletion and aconitase inactivation, ROS over-generation, and lipid peroxidation

**Table 3:** A summary of studies investigating resveratrol effects on muscle injury, fatigue, and damage

References	Exercise modality	Treatment use	No. of subjects	Population of interest	Treatment duration	outcomes
[108]	Short-term downhill training	25 and 50 mg/kg	Control:6 EX:6 EX + RES25: 6 EX + RES150: 6	Mice	4 weeks	High-dose resveratrol prolonged the time before exhaustion and decreased TNF-α mRNA expression and enhanced the mRNA expressions of SIRT1, GLUT4, AMPK α1, and AMPK α2
[109]	Plyometric exercise	500 and 1000 mg/day	Placebo (*n* = 12), RES-500 (*n* = 12), or RES-1000 (*n* = 12)	Untrained males	7 days	Improving recovery, relative mean power, and fatigue index and decreasing injury
[110]	Treadmill	150 mg/kg/day	Control:10 Trained:10 Resveratrol:10	Male rats		Resveratrol is associated with neurotransmitter transport and synaptic vesicle, and the upregulation of KEGG pathways including synaptic vesicle cycle, nicotine addiction, retinol metabolism, insulin secretion, retrograde endocannabinoid signaling, and glutamatergic synapse
[123]	High-intensity exercise	250 mg	–	Healthy, physically inactive men (60–72 years old)	8 weeks	25% reduction in total acetylation level in skeletal muscle

### Curcumin

Curcumin is an FDA-approved *Curcuma longa* extract. Turmeric, which originates from China and India contains three curcuminoid compounds: curcumin (diferuloylmethane), demethoxycurcumin, and bisdemethoxycurcumin, but these compounds make up about 3.14% of the spice. These substances, often called ‘curcumin,’ are present in supplements available for purchase, which usually contain 77% diferuloylmethane, 17% demethoxycurcumin, and 6% bisdemethoxycurcumin [91]. Earlier human studies investigating these effects have administered doses between 90 and 6000 mg per day, with 90 mg being the smallest effective dosage. However, given the data from these studies, the appropriate dosage might be specific to the particular formulation and the bioavailability of the curcumin within the compound [91]. Both antioxidative and anti-inflammatory properties of curcumin have allowed this agent to decrease muscle injury in many ways. The most recent study in this field is conducted in 2023 on humans [92]. Liu and colleagues used curcumin supplementation on middle and high school athletes engaged in wrestling, soccer, and soft tennis. In a 12-week daily exercise training, the participants were assigned to two groups: curcumin group which received a daily dose of curcumin and control group which did not receive any curcumin [92]. For determining muscle damage, they assessed the levels of CK, MDA, 8-OHdG, and TNF-α in the participants’ urine samples and detected that in curcumin group, a notable decline in urinary levels of 8-OHdG was seen after 12 weeks, with the average value dropping from 4.79 to 3.86 ng/mg CRE. However, there were no significant changes in the levels of other markers including CK, MDA, and TNF-α. In the group that received curcumin, both the muscle fatigue score and the muscle soreness score significantly dropped after 12 weeks of supplementation. A significant difference in reaction time, muscle fatigue score, muscle soreness score, and the percentage change in both muscle fatigue and soreness scores were observed between the groups [92]. However, these findings were only detected in male participants [92]. In another study in 2023, the effects of curcumin on muscle fatigue were compared with caffeine. In this study, mice were randomly divided into 3 groups according to their body weight (the control group, the curcumin treatment group, and the caffeine treatment group) and then were given CUMINUP60^®^ (Chenland Nutritionals, Inc., powder, 58 mg·kg-1, dissolved in purified water) or 6mg/kg of caffeine dissolved in purified water. It was observed that both the groups given caffeine and curcumin displayed notable enhancements in exercise-related fatigue when compared to the control group. This was indicated by a longer duration before exhaustion, along with elevated quadriceps coefficients, increased muscle glycogen (MG) levels, and a rise in the expression of the proteins Akt, AMPK, PI3K, and mTOR. Meanwhile, the curcumin group also showed substantial improvements in the exercise fatigue of the mice, highlighted by reduced AMP/ATP ratios and lactic acid (LA) levels, as well as increased glycogen synthase (GS) compared to the caffeine group [93].

In a study in 2022, Chen et al. [94] categorized Male C57BL/6J mice randomly into six groups: blank control (Rest), swimming control (Con), Vitamin C (Vc), low-dose curcumin (C50), middle-dose curcumin (C100), and high-dose curcumin (C200). After 4 weeks of intervention, their investigations revealed that curcumin increases the exercise capacity of mice during the strenuous swimming test. Specifically, the swimming duration for mice in the C100 group increased by 273.5% compared to the Con group. Through analyzing gene expression and protein levels they found out that CUR reduced oxidative stress caused by exercise and notably improved the activities of superoxide dismutase, catalase, and glutathione peroxidase by stimulating the Nrf2 signaling pathway [94].

Another study in 2015, tried different doses of curcumin (180 μg/mL by oral gavage at 0, 12.3, 24.6, or 61.5 mL/kg/day for 4 weeks) on male ICR mice and assessed the levels of their serum lactate, ammonia, blood urea nitrogen (BUN), and glucose and tissue damage markers such as aspartate transaminase (AST), alanine transaminase (ALT), and creatine kinase (CK) after forelimb grip strength, exhaustive swimming [95]. They detected that endurance in exercise varied considerably depending on the CUR treatments. The swimming duration was significantly increased by 1.98, 2.17, and 2.22 times with CUR-1X, CUR-2X, and CUR-5X treatments, respectively, compared to the vehicle treatment. They also observed that the levels of serum lactate and ammonia are also different among the divided groups. Lactate level was decreased by CUR treatment, by 33.5%, 36.7% and 40.5%, with CUR-1X, CUR-2X, and CUR-5X treatment, respectively. When compared to placebo, groups that received CUR had lower CK level by 45%, 52% and 60% (all *p* < 0.001), with CUR-1X, CUR-2X, and CUR-5X treatments, respectively. Furthermore, AST level was also significantly lower by 21% to 31%. The effects of curcumin on ALT levels were the same as AST. Other than that liver glycogen level did not differ after CUR treatment while muscle glycogen level significantly increased by 1.39- to 1.49-fold with CUR supplementation compared to vehicle treatment [95]. An older study applied their study to anti-inflammatory effects of curcumin through examining IL-1beta, IL-6, TNF-alpha, and creatine kinase in mice after two sets of treadmill running. They recognized that downhill running led to a reduction in treadmill run time until fatigue (at both 48 and 72 h) and voluntary activity (at 24 h). However, the inclusion of curcumin in the diet mitigated these negative impacts on running performance. Additionally, downhill running was linked to elevated levels of inflammatory cytokines (at 24 and 48 h) and creatine kinase (at 24 h) (P < 0.05), but these increases were reduced by curcumin supplementation [96].

### Quercetin

Quercetin (QUE) is derived from quercetum (oak forest) and is considered to be a plant flavonol from the flavonoid group of polyphenols. There are several fruits, vegetables, leaves, seeds, and grains which this agent can be found in. Many studies have used this natural compound for decreasing muscle damage and fatigue. One of the first studies which used QUE is conducted in 2007 by Neiman et al. [97] on 63 runners. Participants in the study were randomly assigned to either the quercetin group or the placebo group, and using double-blind methods, they consumed 1000 mg of quercetin daily for a duration of 3 weeks before a 160 km race. In their analysis, no significant differences were detected in CRP, IL-6, Ck, and cortisol levels as well as no significant difference measured for leukocyte IL-8 IL-1Ra IL-10 [97]. The results of this study shows that this amount of quercetin cannot make any difference in muscle damage after running [97]. Another study on humans is performed by Patrizio et al. [98] on 10 young men. In this randomized, double-blind, crossover study, participants took QUE (1 g/day) or a placebo (PLA) three hours before a resistance training session. This session included three sets of eight repetitions at 80% of their one repetition maximum for eight different resistance exercises, performed using both sides of the bod [98]. After a single dose of QUE, the torque–velocity curve for knee extensors improved. Following resistance exercise, participants experienced a smaller decrease in maximum voluntary isometric contraction (MVIC) when using QUE compared to the placebo. Additionally, there was a notable increase in the rate of torque development and a rise in the neuromuscular efficiency ratio. The total volume of resistance exercises was significantly higher with QUE than with PLA, while the rating of perceived exertion (RPE) scores was alike [98]. The most recent study on human participants in conducted in 2022 in Taiwan [99]. In this study, 12 healthy physically active students were treated with 1000 mg of QUE per day for 7 days and then they went through exercise consisting of 70% V̇O2max cycling for 60 min, followed by 3 h of recovery, then a subsequent single bout of cycling exercise with 75% V̇O2max to exhaustion [99]. They observed that QUE reduced the insulin response triggered by glucose after exercise, boosted total antioxidant capacity (TAC) and superoxide dismutase (SOD) activities, and lowered malondialdehyde (MDA) levels during the recovery phase [99]. After quercetin treatment, cycling performance at 75% of V̇O2max significantly improved, and there were reduced levels of interleukin 6 and creatine kinase after 24 h. However, there were no notable differences in glucose, respiratory exchange rate, TNF-α, myoglobin, or high sensitivity C-reactive protein when comparing quercetin and placebo trials [99]. Another study on 26 badminton players showed paradoxical results. According to this study, after 8 weeks of quercetin administration, there were no notable differences in lactate concentration, body fat percentage, or VO2 max either between the two groups or within a single group after 8 weeks of supplementation with either a placebo or quercetin. However, the quercetin group experienced a significant rise in time to exhaustion (TTE) after the intervention (*P* < 0.05), while the placebo group did not show any significant changes [100].

In muscle recovery point of view, Bazzucchi et al. [101] aimed to examine if QUE could enhance the recovery of neuromuscular function and biochemical indicators in the week following muscle damage caused by eccentric exercise (EEIMD). They examined 16 men who took either Q (1000 mg/day) or a placebo (PLA) for 14 days as part of a double-blind crossover study. A neuromuscular (NM) assessment was conducted before and after the exercise, as well as at 24 h, 48 h, 72 h, 96 h, and 7 days following intense eccentric exercise. The relationship between force and velocity of the elbow flexor muscles, along with their maximal voluntary isometric contraction (MVIC), was recorded simultaneously with electromyographic signals (EMG). 16 men took either QUE (1000 mg/day) or a PLA for 14 days as part of a double-blind crossover study. A NM assessment was conducted before and after the exercise, as well as at 24 h, 48 h, 72 h, 96 h, and 7 days following intense eccentric exercise. Supplementing with QUE for 14 days appears to enhance recovery from weakness caused by eccentric exercise [101]. Another placebo-controlled, double-blind clinical trial on 60 men also shows that CK levels as a marker for muscle injury is reduced in participants who received QUE [102]. A study in 2020 tried to examine the combination of QUE with a mango leaf extract and compare their effects in men and women [103]. The used the combination of QUE and a mango leaf extract on 24 women and 33 men 24 h before an exercise program containing 5 km running performance and ran a 10 km race followed by 100 drop jumps [103]. The results of this study shows that mechanical impulse, loss of jumping performance, and muscle pain are three important factors that can be decreased by QUE supplementation [103]. For a better understanding of the underlying mechanisms of the mentioned effects, a group of researchers examined IGF-I and IGF-II levels in men who received 1g/day of QUE before an eccentric-induced muscle-damaging protocol [104]. They observed that in PLA group, muscle damage markers including CK, LDH, and myoglobin along with IGF-I and IGF-II were increased while QUE treatment reversed these effects. Following QUE supplementation, there was a significant rise in IGF-I levels, and it was particularly observed that the peak of IGF-II occurred earlier compared to the placebo, coinciding with the same time as IGF-I (72 h). They also declared that decreasing the levels of IL-6 is one of the interesting effects of QUE which enhances post-exercise recovery [104].

An animal study tried to confirm the antioxidant effects of QUE on muscle damage. They observed that after QUE treatment, strenuous exercise in mice led to a rise in the leakage of creatine kinase-MB (increased from 221.5 ± 33.8 to 151.1 ± 19.1 U/l, *P* < 0.01) and caused significant damage to their muscle structure, particularly noted by damaged myofibrils and enlarged mitochondria. This damage was considerably reduced with the use of quercetin as a preventive treatment [105]. QUE pretreatment significantly reduced mitochondrial oxidative stress by preventing the depletion of glutathione, inactivating aconitase, excessive production of ROS, and lipid peroxidation in the heart mitochondria of mice subjected to intense exercise. Additionally, mitochondrial dysfunction was indicated by a reduction in mitochondrial membrane potential (*P* < 0.01) and a lower respiratory control ratio (*P* < 0.01) as a result of acute exercise [105]. Another animal trial was conducted in 2014 on 4 groups of rats (containing quercetin sedentary, quercetin exercised, placebo sedentary, and placebo exercised) [106]. In this study, treadmill training was conducted five times a week for 6 weeks. The groups receiving quercetin were given this supplement via gavage every other day during the study. Measurements were taken for Sirtuin 1 (SIRT1), mRNA levels of peroxisome proliferator-activated receptor γ coactivator-1α, mitochondrial DNA (mtDNA) content, and citrate synthase (CS) activity in the quadriceps muscle [106].

### Resveratrol

Resveratrol (RSV) is a polyphenolic stilbene characterized by a double bond that links two phenol rings, appearing as a white powder. This bond facilitates geometric isomerization when exposed to UV light. The trans isomer is not only more biologically active, but also more prevalent than the cis isomer [107]. The chemopreventive effects of resveratrol have been researched for many years. Although it holds promise for improving cancer therapy, the compound has certain pharmaceutical drawbacks, including a poor pharmacokinetic profile and low bioavailability. According to both in vivo and in vitro studies, 500 mg of RSV is well-tolerated and well-absorbed. There are plenty of studies investigating the effects of RSV on exercise-induced muscle damage. The most recent study in this field is performed on 24 mice aged 6 weeks [108]. In this study, mice were categorized into 4 groups according to the amount of resveratrol intake: control, exercise, exercise with low-dose resveratrol (25 mg/kg body weight), and exercise with high-dose resveratrol (150 mg/kg body weight) [108]. The primary outcomes of this study show the duration until exhaustion for the exercise group was less than that of the control group; however, the exercise and low-dose RSV groups showed no difference in the time before exhaustion. A notable difference was observed between the exercise and high-dose RSV groups. Regarding muscle damage, in comparison to the control group, both LDH and CK levels were significantly higher in the EX + RES25 and EX + RES150 groups. However, the levels of LDH and CK in the EX + RES150 group were notably lower than those observed in the EX-group [108]. In the EX-group, the mRNA expression of TNF-α in the gastrocnemius muscle was significantly higher than in both the control group and the EX + RES150 group. The results indicated that the mRNA levels of SIRT1, AMPK α1, and AMPK α2 in the gastrocnemius muscle for the EX + RES25 group were notably greater than those in the EX-group. Additionally, the mRNA expression levels of GLUT4, AMPK α1, and PGC-1α in the gastrocnemius muscle of the EX + RES150 group were also significantly elevated compared to the EX-group. There was a significant difference in the mRNA expression of SIRT1 and PGC-1α between the EX + RES25 and EX + RES150 groups [108]. In conclusion, a high-dose resveratrol treatment extended the duration before reaching exhaustion during brief downhill sprints. This treatment also led to reduced levels of TNF-α mRNA and increased levels of SIRT1, GLUT4, AMPK α1, and AMPK α2 mRNA in certain muscles. These findings suggest that high-dose resveratrol supplementation may help diminish inflammation and oxidative stress while enhancing energy utilization during short bursts of intense exercise [108].

A similar study also examined markers of muscular damage in 36 men after a Plyometric exercise. They detected that At 72 h after exercising-induced muscle damage (EIMD), the peak force (FP) and rate of force development (RFD) during the counter movement jump (CMJ) in the resistance training groups were not significantly different from the baseline measurements, yet they were substantially higher than those in the placebo group [109]. The RSV group showed improved recovery effects on relative peak power (RPP), relative mean power (RMP), and fatigue index (FI), especially among those in the high-dose group (*p* < 0.05). In terms of muscle pain following PEIMD, the group receiving RSV supplements had significantly better outcomes compared to the placebo group (*p* < 0.05). Furthermore, regarding muscle damage indicators like creatine kinase (CK) and lactate dehydrogenase (LDH) after PEIMD, RSV supplementation significantly reduced levels and sped up recovery (*p* < 0.05) [109]. An animal study tried to identify the genes which their expression can be different after RSV administration. They showed that the top enriched GO terms in the trained group compared to the control group were mainly related to RNA metabolic processes and transmembrane transporters. Additionally, the KEGG pathways that showed significant increases encompassed mucin-type O-glycan biosynthesis, drug metabolism, and pyrimidine metabolism. In contrast, the most enriched GO terms in the resveratrol group against the control group were primarily linked to neurotransmitter transport and synaptic vesicles, with upregulated KEGG pathways including the synaptic vesicle cycle [110].

### Other polyphenols

Besides the mentioned polyphenols which are the most studied ones in this field, there are also some other types of polyphenols which are examined on athlete for clarifying their effects on muscle damage. For instance, a study aimed to explore the effects of acacia polyphenol (AP) supplementation on exercise-induced oxidative stress in mouse liver and skeletal muscle [111]. After exhaustive exercise, plasma aspartate aminotransferase (AST) levels rose, along with an increase in thiobarbituric acid reactive substances (TBARS) in the liver and skeletal muscle, indicating oxidative stress and tissue damage. Additionally, exercise resulted in a decline in liver GSH levels. Surprisingly, AP supplementation led to elevated plasma AST and alanine aminotransferase levels, increased liver TBARS, and heightened protein carbonyl levels, suggesting it might enhance oxidative stress and hepatotoxicity in the liver. Furthermore, AP reduced both GSH and glutathione peroxidase activity in the liver, further indicating potential liver damage [111]. Conversely, the study found that AP supplementation significantly decreased TBARS levels in skeletal muscle, suggesting a protective effect against oxidative stress in this tissue. In summary, while high-dose AP potentially mitigates oxidative stress in skeletal muscle, it appears to exacerbate oxidative stress and toxicity in the liver, highlighting the dual effects of AP supplementation depending on the tissue type involved. This nuanced understanding of AP's impact is critical for its potential therapeutic applications [111].

Another study established a model of skeletal muscle injury in mice caused by excessive exercise and utilized gallic acid as an intervention [112]. To measure markers of muscle damage and indicators related to ferroptosis, various techniques including ELISA, Western blot, and RT-qPCR were employed to evaluate levels of CK, LDH, IL-6, TNF-α, Fe2 + , MDA, COX2, and GPX4. The findings indicate that GA has beneficial effects on inflammation and injury in skeletal muscle caused by excessive exercise. GA mitigated mitochondrial damage and redox imbalance by lowering membrane potential and enhancing ATP production. Furthermore, GA also prevented ferroptosis in skeletal muscle cells due to its antioxidant properties and ability to reduce iron accumulation [112]. Overall, GA shows promise as a therapeutic agent for protecting against skeletal muscle injury from excessive exercise by targeting mitochondrial oxidative stress and ferroptosis pathways [112].

After all, some argue that the findings from experiments on animals are not applicable to humans due to the biological variations between species and because the outcomes can vary based on the specific animal model used and therefore, it is necessary to gather clinical trials in this field for a definite conclusion about the efficacy of polyphenols in post-exercise muscle damage.

## Clinical trials

The most recent human study in this field is conducted in 2024 by Valder et al. [113] which explores the impact of short-term supplementation with chokeberry juice compared to a placebo on muscle damage, oxidative stress, and leg strength in recreational endurance athletes undergoing a 6 day high-intensity interval training (HIIT) protocol. In this study, 18 recreational athletes participated in a crossover design, where they received either chokeberry juice or a placebo drink. They underwent baseline assessments, including blood samples to measure markers of muscle damage and oxidative status, as well as leg strength measurements both before and after the HIIT protocol [113]. Both groups experienced significant muscle damage post-exercise, indicated by increases in creatine kinase (CK) levels (*p* = 0.001). No significant differences were found between the juice and placebo groups regarding exercise-induced muscle damage (*p* = 0.371) or oxidative status (*p* = 0.632). Leg strength reduction was observed, with the placebo group showing a greater decline in strength (*p* = 0.988), though this also lacked statistical significance. Taken together, this study concludes that while no significant beneficial effects of chokeberry juice on muscle damage or oxidative status were observed, there was a trend suggesting less strength reduction in the juice group compared to the placebo. However, the authors caution that these potential effects might require longer supplementation periods or higher polyphenol concentrations. Further studies are warranted to verify these findings and explore the implications of chokeberry juice supplementation in athletic recovery [113].

Corr and colleagues conducted a study in 2020 on Cocoa flavanols (CF) [114]. Their study explored the effects of CF on recovery from exercise-induced muscle damage. This randomized, single-blind trial involved 23 participants (13 females, 10 males) divided into three groups: a control group receiving 0 mg CF (*n* = 8), a high dose of 830 mg CF (CF830, *n* = 8), and a supra dose of 1245 mg CF (CF1245, *n* = 7). After undergoing a muscle damage protocol consisting of maximal concentric/eccentric hamstring curls, participants consumed their assigned drink. Recovery was assessed through maximal voluntary isometric contraction (MVIC) of knee flexors at two angles, as well as visual analog scale (VAS) and lower-extremity function scales measured at baseline, and 24, 48, and 72 h post-exercise. While a significant time effect was noted for all measures (*p* < 0.05), no significant differences emerged between the groups (*p* ≥ 0.17). At 48 h, there were large effect sizes in favor of the CF1245 group compared to control for several measures, but this was not statistically significant. Consequently, this study concludes that acute consumption of cocoa flavanols does not provide a beneficial impact on muscle recovery [114].

Another previous research has explored the antioxidant and anti-inflammatory benefits of green tea compounds in various human tissues, with positive effects noted in the brain. However, it was unclear if similar benefits would extend to skeletal muscle [115]. This study aimed to investigate whether green tea extract could alleviate exercise-induced muscle soreness, muscle damage, and oxidative stress. They conducted a randomized, double-blind, placebo-controlled trial involving 20 untrained men who underwent exercise sessions to induce delayed-onset muscle soreness in the triceps sural muscle. They were given either 500 mg/day of green tea extract (*n* = 10) or a placebo (*n* = 10) for 15 days. Muscle soreness was assessed using a visual scale, and blood samples were collected at various points to evaluate markers of muscle damage, oxidative stress, and antioxidant levels. The results indicated that while exercise did lead to muscle soreness, the supplementation with green tea extract reduced muscle damage but did not impact the sensation of soreness. Additionally, there were no significant effects on plasma markers of oxidative damage or antioxidant status. In summary, while green tea extract did not alleviate delayed-onset muscle soreness, it appeared to aid in reducing muscle damage, suggesting it may support recovery after intense exercise [115].

Ther are also some clinical trials which have tried CUR on human subjects. In a study by Tanabe et al. [116], influence of CUR intake on muscle damage and inflammatory responses related to exercise, with different timing of ingestion was examined. Conducted as a double-blind crossover with parallel experiments, the first part involved ten healthy men consuming 180 mg of CUR or a placebo for seven days prior to exercise. The second part had another group of ten men consuming CUR or placebo for seven days following exercise. Participants performed 30 maximal eccentric contractions of the elbow flexors, with assessments of maximal voluntary contraction (MVC) torque, elbow range of motion (ROM), muscle soreness, and serum creatine kinase (CK) levels measured before, immediately after, and across several days’ post-exercise [116]. Plasma interleukin-8 (IL-8) levels were also evaluated at various intervals. In the first experiment, no significant differences in muscle parameters were observed between CUR and placebo, but inflammation (IL-8) was notably lower 12 hours’ post-exercise when CUR was taken beforehand. Conversely, in the second experiment, CUR supplementation after exercise led to better MVC torque and ROM (improved at 3–7 days and 2–7 days’ post-exercise, respectively), while also reducing muscle soreness and CK levels (lower at 3–6 days and 5–7 days’ post-exercise). Therefore, taking CUR before exercise may help reduce acute inflammation, while taking it afterward can lessen muscle damage and promote quicker recovery [116].

Tanabe and colleagues also conducted a similar study in 2024 which investigated whether curcumin supplementation could reduce muscle damage, soreness, and inflammation in collegiate soccer players after a match, given previous evidence that curcumin had such effects in laboratory and field studies. Fifteen athletes from the same college team participated in a randomized, double-blind, cross-over study where they consumed either 180 mg of curcumin or a placebo one hour before the match and for two days afterward. Muscle soreness, jump performance, and various muscle damage markers were assessed before and after the match. The results indicated no significant differences in recovery markers between the curcumin and placebo groups, suggesting that curcumin does not aid recovery from muscle damage post-soccer match in these athletes [117].

Another study was also performed in 2020 by a group of researchers who investigated the effects of curcumin supplementation (1.5 g/day) on exercise-induced oxidative stress, inflammation, muscle damage, and soreness in 19 males [118]. In this randomized, double-blinded, placebo-controlled trial, participants underwent a muscle-damaging protocol before and after 28 days of supplementation. Blood samples were analyzed for total antioxidant capacity, malondialdehyde, tumor necrosis factor alpha, and creatine kinase, while perceived muscle soreness was measured using a visual analog scale. Results showed that curcumin significantly reduced creatine kinase levels (199.62 U/L) compared to the placebo (287.03 U/L) and decreased muscle soreness (VAS 2.88 vs. 3.36, *p* = 0.0120). However, no significant differences were observed in total antioxidant capacity, TNF-α, or malondialdehyde levels. The findings suggest that curcumin may alleviate muscle damage and soreness without negatively affecting the natural inflammatory response post-exercise. Future research should explore the long-term effects of curcumin on muscle recovery [118].

There are also some studies which specifically investigated the effects of CUR on muscle soreness. For instance, Abbott et al. [119] used 500 mg of CUR or a control substance on 11 players from an English Premier League under-23 team after a 90 min match. These researchers measured countermovement jump height (CMJ), reactive strength index (RSI), delayed-onset muscle soreness (DOMS), and subjective well-being at various post-match intervals. Results showed no significant differences in external load or dietary intake between the two conditions. However, CURC significantly improved CMJ and RSI performance and reduced DOMS at all measured times post-match, with the most significant benefits observed 12 and 36 hours afterward. The findings indicate that CURC may help professional soccer players recover from muscle soreness and function deficits following a match [119].

Mallard et al. [120] also found that using CUR leads to lower post-exercise capillary lactate levels (7.4 mmol/L) compared to the placebo group (8.8 mmol/L). Additionally, in their study, the placebo group reported higher overall muscle pain at 48 and 72 h post-exercise. Curcumin also reduced total creatine (TC) levels compared to placebo at 24 and 48 h post-exercise. These results suggest that curcumin may help athletes recover more quickly, endure higher training intensity, and reduce post-exercise pain and inflammation [120].

Another study used Cureit^™^—a bioavailable form of curcumin and found out that oral consumption of Cureit significantly reduced DOMS, slightly lowered creatinine kinase levels, and mildly increased VO2 max compared to a placebo, with no side effects noted [121]. These results indicate that Cureit promotes better recovery and lessens DOMS, attributed to the enhanced bioavailability of curcumin [121].

## Conclusion and future prospective

As mentioned, polyphenols have many beneficial effects that are practical for the prevention and/or treatment of many diseases. Here we have reviewed the studies which examined different types of polyphenols on either humans or animals after one or more sessions of training. Curcumin is one of the most studied polyphenols in this field that is shown to be effective on decreasing muscle damage and fatigue, mostly through decreasing the levels of oxidative stress. The most observed curcumin’s mechanisms of action are activating Nrf2 signaling pathway and PI3K/Akt/AMPK/mTOR pathway in muscle cells. Quercetin is also well-studied in this field and there are plenty of human studies which have approved its roles in decreasing muscle injury and increase the time before exhaustion; however, not much evidence has been provided for clarifying the underlying mechanisms of quercetin’s actions. Resveratrol is also confirmed to be well-tolerated and well-absorbed and have the best efficacy at 500 mg/kg dosage. From the muscle injury point of view, it is shown that RSV exerts its effects mainly through reducing acetylation levels and dysregulating some genes linked to neurotransmitter transport and synaptic vesicles.

In conclusion, the growing body of research indicates that polyphenols play a significant role in mitigating post-exercise muscle damage, underscoring their potential as a valuable nutritional strategy for athletes and fitness enthusiasts alike. These naturally occurring compounds, found abundantly in fruits, vegetables, tea, wine, and other plant-based foods, exhibit potent antioxidant and anti-inflammatory properties. This dual action is crucial in managing oxidative stress and inflammation, both of which are exacerbated by intense physical activity and contribute to muscle soreness and damage.

Studies have shown that the consumption of polyphenol-rich foods or supplements before or after exercise can lead to reduced markers of muscle damage, such as creatine kinase levels, and a decrease in delayed-onset muscle soreness (DOMS). The impact of specific polyphenols, such as quercetin, catechins, and anthocyanins, has been particularly notable in promoting muscle recovery and enhancing exercise performance. Furthermore, the mechanisms through which polyphenols exert their protective effects include modulating inflammatory pathways, enhancing muscle repair processes, and improving mitochondrial function, thus supporting overall muscle health.

However, while the evidence is promising, further research is necessary to fully understand the optimal types, doses, and timing of polyphenol intake for recovery purposes. Variability in individual responses, the bioavailability of different polyphenols, and the influence of dietary patterns on their effectiveness require careful consideration in future studies. It is also important to recognize that polyphenols should be viewed as a complementary approach rather than a standalone solution, integrated into a broader recovery strategy that includes proper hydration, nutrition, rest, and active recovery methods.

In summary, the incorporation of polyphenols into an athlete’s diet may serve as a practical and effective means of reducing post-exercise muscle damage and enhancing recovery. As more insights emerge from ongoing investigations, the potential of these compounds to improve athletic performance and overall health will become increasingly clearer, paving the way for their strategic use in exercise nutrition regimens.

## Data Availability

No datasets were generated or analysed during the current study.
